# Is “Preparation for Oxidative Stress” a Case of Physiological Conditioning Hormesis?

**DOI:** 10.3389/fphys.2018.00945

**Published:** 2018-08-02

**Authors:** Marcus F. Oliveira, Marcio A. Geihs, Thiago F. A. França, Daniel C. Moreira, Marcelo Hermes-Lima

**Affiliations:** ^1^Instituto de Bioquímica Médica Leopoldo de Meis, Universidade Federal do Rio de Janeiro, Rio de Janeiro, Brazil; ^2^Programa de Pós-Graduação em Ciências Fisiológicas, Instituto de Ciências Biológicas, Universidade Federal do Rio Grande, Rio Grande, Brazil; ^3^Área de Morfologia, Faculdade de Medicina, Universidade de Brasília, Brasilia, Brazil; ^4^Departamento de Biologia Celular, Instituto de Ciências Biológicas, Universidade de Brasília, Brasilia, Brazil

**Keywords:** antioxidant, biochemical adaptation, estivation, hypoxia, oxidative stress, reactive oxygen species, redox

## Introduction

Many animal species endure hypoxic or even anoxic stresses, when faced with harsh environmental conditions including freezing, severe dehydration and air exposure of aquatic organisms. Hypoxia in those animals induces a set of physiological/biochemical adaptive responses, allowing organisms to cope with low oxygen levels. Such responses are mediated by (i) arrest of transcriptional and translational activity, (ii) depression of metabolic rate, (iii) re-wiring of energy metabolism pathways toward fermentative rather than oxidative routes, (iv) activation of mechanisms involved in both macromolecular repair and detoxification of cellular-derived oxidants (Storey and Storey, 2011; Storey, 2015). In this regard, a transient up-regulation of endogenous antioxidant enzymes aiming the improvement of reactive species (RS) detoxification has emerged as a hallmark for many organisms to tolerate hypoxic stresses. Such phenomenon was coined “preparation for oxidative stress” (POS) 20 years ago, and numerous examples have supported POS as a physiological mechanism to deal with environmental stresses (Hermes-Lima and Storey, 1995, 1996; Hermes-Lima et al., 1998, 2015; Hermes-Lima and Zenteno-Savín, 2002; Lushchak et al., 2005; Welker et al., 2013). So far, we have identified POS as an adaptive physiological mechanism in 83 animal species from 8 different phyla when exposed to low oxygen stress and during estivation (Moreira et al., 2016, 2017). The phenotypes generated by POS include the up-regulation of superoxide dismutase (SOD), catalase and glutathione transferase (GST) activities by ~80% in *Otala lactea* snails during estivation (Hermes-Lima and Storey, 1995). Interestingly, snails that return to active state decrease all antioxidant enzyme activities to pre-estivation levels. Similar observations were reported when *Rana pipiens* frogs were challenged with 30 h anoxia, causing transient catalase, and GST activation (Hermes-Lima and Storey, 1996). Also, transient increases of catalase and glutathione peroxidase (GPX) activities by 30–70% were observed in the brain of common carp during hypoxia (Lushchak et al., 2005). Increases by ~60% in muscular SOD activity were also observed in *Lacerta vivipara* lizards upon freezing, which returns to control levels after thawing (Voituron et al., 2006).

**Figure 1:**
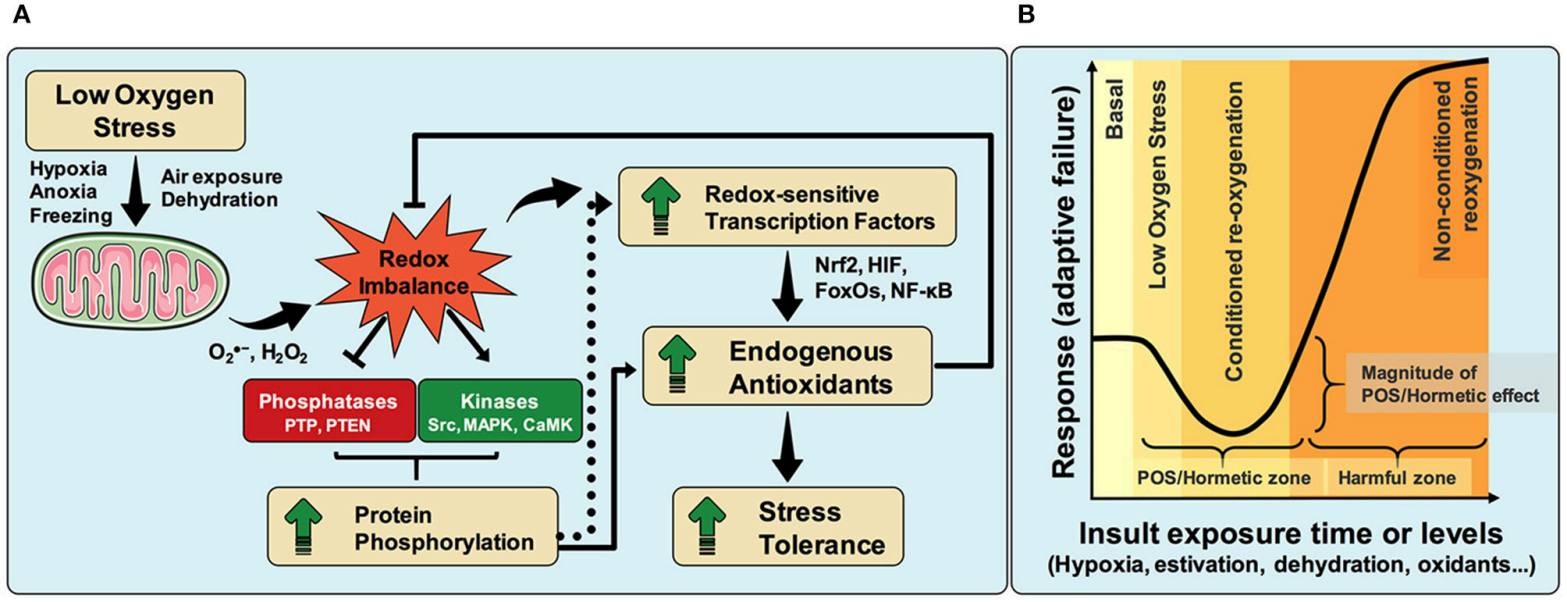
Molecular oxygen is absolutely required for maintenance of cellular energy and redox homeostasis across different animal species. Although some organisms cannot tolerate slight hypoxia, others can adapt to and survive strong shortages in oxygen supply even for long periods of time. A common trend observed in some hypoxia-tolerant animals is their enhanced capacity to boost antioxidant defenses during a number of stresses, a phenomenon known as “preparation for oxidative stress” (POS). POS was identified in animals from 8 distinct phyla and despite the molecular mechanisms are not fully understood, we have recently proposed an explanation (Hermes-Lima et al., 2015), where the role of phosphatases and kinases in POS is highlighted herein, as well as the increased cellular oxidant production under hypoxia **(A)**. During hypoxia, the redox state of ETS and mitochondrial dehydrogenases shifts toward a reduced state due to limited electron transfer from cytochrome *c* oxidase to oxygen. This leads to increased electron availability in many enzymes/complexes involved in redox reactions, consequently favoring superoxide production (Smith et al., 2017). Importantly, given that a very small percentage of molecular oxygen is converted to superoxide in isolated mammalian mitochondria (about 0.2%, Tahara et al., 2009) and that this figure is likely to be much lower *in vivo* (Murphy, 2009), it is suggestive that the electron availability, not oxygen concentration, would be the limiting factor in mitochondrial superoxide production (Campian et al., 2007). Therefore, even in hypoxia, increases in electron availability should boost mitochondrial superoxide production—at least until molecular oxygen concentration becomes so low that electron availability ceases to be the limiting factor. Thus, the overall pattern observed is an increase in oxidant formation during hypoxia. The pattern of transient activation of antioxidant defenses along hypoxic challenges, and the improved protection against stressful insults generated afterwards, follows the same trend observed in many cases of physiological conditioned hormesis. Limited time and magnitude exposure of animals to insults including hypoxia/anoxia, freezing and severe dehydration, as well as to conditions inducing estivation, activates a “physiological program” that reduces adaptive failure and/or mortality upon stronger challenges (the “hormetic zone”), as proposed in the hormesis concept. “Conditioned re-oxygenation” (or reoxygenation-like, during dehydration/rehydration and freezing/thawing), shown in **(B)**, is a state where the protective POS-response range is maximum. However, longer and/or stronger exposure to these insults revert the protective hormetic effects (the “harmful zone”), increasing adaptive failure. Therefore, given their remarkable similarities in biological and biochemical outputs, we propose that POS should be included as a new example of physiological conditioning hormesis. Graphic elements adapted from Servier Medical Art.

Evidence suggests the existence of common mechanisms underlying dormancy states induced by hypoxia, hypoxic-like conditions and aerobic hypometabolism. For example, it is known that hypoxia maintains the redox state of mitochondrial electron transport system (ETS) toward a reduced state, favoring the production of superoxide radicals (Chandel et al., 1998; Vanden Hoek et al., 1998; Hernansanz-Agustín et al., 2014). Thus, against the common-sense, reduced oxygenation increases, rather than decreases, cellular oxidants production (Murphy, 2009; Smith et al., 2017; see legend of Figure 1A). Accordingly, the proposed mechanism by which POS confers tolerance to oxidant insults, considers an increase in mitochondrial RS formation during low oxygen stress, followed by redox imbalance that activates redox-sensitive transcription factors, such as NF-κB, FoxOs, and Nrf2 (Schreck et al., 1991; Ishii et al., 2000; Essers et al., 2004). Additionally, redox imbalance also shifts protein phosphorylation levels toward a higher phosphorylated state, by either reducing protein phosphatase and/or increasing protein kinase activities (Staal et al., 1994; Meng et al., 2002; Howe et al., 2004; Corcoran and Cotter, 2013) (Figure 1A). In this regard, oxidants can inhibit multiple protein tyrosine phosphatases including PTP1B and PTEN (Leslie et al., 2003; Salmeen et al., 2003), with direct consequences to cell function. Conversely, oxidant conditions activate several protein kinases such as Src (Devary et al., 1992), MAPK (Goldstone and Hunt, 1997) and calcium/calmodulin-dependent protein kinases (Howe et al., 2004). However, it seems that maintenance of the higher phosphorylated state of protein targets by redox imbalance may occur through protein phosphatase inhibition rather than direct protein kinase activation by oxidants (Lee and Esselman, 2002). The consequences of higher protein phosphorylation to cellular redox homeostasis are: (i) the activation of redox-sensitive transcription factors (Shirakawa and Mizel, 1989), and/or (ii) regulation of antioxidant enzymes activities by direct phosphorylation. Examples include the demonstration that Nrf2 expression depends on low PTEN phosphatase activity, rendering tumor cells more proliferative (Rojo et al., 2014). Likewise, maintenance of oxidant conditions indirectly activates antioxidant enzymes through their phosphorylation, acting independently of redox-sensitive transcription factors (Rhee and Woo, 2011; Rafikov et al., 2014; Tsang et al., 2014). Ultimately, higher tolerance to multiple redox stresses is afforded by increasing endogenous antioxidant levels mediated by either activation of redox-sensitive transcription factors or by activation of antioxidant enzymes through phosphorylation or other covalent modifications (Figure 1A).

## Examples of POS-adaptation strategy

Two recent studies provide good examples of natural strategies involved in POS-adaptation. The first study demonstrated that expression of SOD, catalase, and GST in the mussel *Mytilus galloprovincialis* significantly increases after 8–24 h of aerial exposure (Giannetto et al., 2017). Interestingly, the expression level of all antioxidant enzymes returns to baseline levels during re-submersion. This observation strongly indicates that under low oxygen stress, this mussel enhances antioxidant defenses to cope with the redox burst during re-oxygenation. In a second study, the expression/activity of several antioxidant enzymes and oxidative stress markers increased in the liver of *Pelteobagrus vachelli* fish upon hypoxic challenge and declined to control values following re-oxygenation (Zhang et al., 2016). Collectively, it seems likely that hypoxia boosts oxidant formation and mild molecular oxidative damage, which ultimately activates the antioxidant defenses. However, direct evidence demonstrating the involvement of redox-sensitive transcription factors and oxidant overproduction under hypoxia remains elusive.

When POS was first proposed, it was thought that the increase in endogenous antioxidants levels was a necessary response of animals under hypoxia exclusively to cope with higher oxidant formation during re-oxygenation. However, further experimental evidence demonstrated that production of oxygen-RS actually increases during hypoxia in different animal models. Thus, hypoxia induces a RS-mediated response that prepares animals to cope with a stronger redox challenge during re-oxygenation. Similar effects were previously reported in hypoxic pre-conditioning in different tissues, which ultimately renders cells more resistant to reperfusion insult (Murry et al., 1986; Vanden Hoek et al., 1998). The antique Paracelsus concept that “the dose makes the poison” (or the antidote, in another version), led us to compare the mechanisms underlying POS, and its biological outputs, with a known concept from toxicology that mediates adaptive cellular and organism responses induced by a mild stress that improves tolerance against stronger challenges.

## Is POS an example of physiological conditioning hormesis?

Although the exact underlying molecular mechanisms of POS remain to be confirmed, the response convergence toward improved antioxidant defenses is strikingly evident across species. Indeed, many cells and organisms exposed to mild sub-lethal stress conditions trigger a protective response against stronger subsequent challenges. Importantly, the magnitude of stress exposure can induce a dose–response effect that results in opposite outputs at low or high stress levels (Calabrese and Blain, 2011). These opposite effects usually manifest as “inverted U-shaped” or a “J-shaped” dose–response curves, which are considered as hallmarks of the so-called hormesis process (Figure 1B) (Southam and Ehrlich, 1943; Calabrese and Blain, 2011).

The stress challenges that trigger hormesis include exposure to pollutants, toxins, natural products, caloric restriction, ischemic pre-conditioning, ionizing radiation, and many other stimuli (Schmitt et al., 2002; Schulz et al., 2007; Calabrese et al., 2012; Schmeisser et al., 2013). Among the beneficial outputs of hormesis, improved survival to stronger chemical challenges, hypoxia, and re-oxygenation, as well as increased longevity were reported (Calabrese et al., 2012). Conceivably, given their remarkable similarities in biological and biochemical outputs, it might well be the case that POS would be included as a new example of physiological conditioning hormesis. If that is the case, it is critical to determine whether the magnitude of environmental stresses in POS studies would induce dose-response patterns that meet the hormesis criteria. These include at least 10% increase (the “inverted U-shaped curve”) or a 3% reduction (the “J-shaped curve”) followed by a return in response to a given stimuli (Calabrese and Blain, 2005). These responses could be a function of exposure time or concentration/intensity of the stimuli.

Because it is a classic stressor that generates POS-response in organisms, studies on the dynamic range of hypoxia intensity can clearly demonstrate the phenomenon of hormesis. In this sense, studies that altered oxygen levels and observed possible oxidative effects on the aquatic biota present classical hormesis profiles. For example, scallops subjected to hypoxic challenges produced a biphasic response for SOD activity, with an early 15–50% activation (at 12 h exposure), followed by up to 40–60% reductions (from 7 to 21 days) (Chen et al., 2007). This pattern of regulation of SOD activity fits well within the hormesis concept. Up-regulation of GST and GPX activities were observed in *Catla catla* carps when exposed to different degrees of hypoxia (Singh et al., 2015). In the pacific white shrimp, clear hormetic responses for SOD and GPX activities were reported upon exposure to different degrees of hypoxia, producing remarkable “inverted U-shaped curves” (Li et al., 2016). Interestingly, in several cases, markers of redox imbalance follow quite closely the trend observed for antioxidant enzymes upon hypoxia and reperfusion, suggesting that macromolecular oxidation might act as mediators of up-regulation of redox defensive mechanisms. Therefore, given that redox metabolism endpoints assessed so far can be included as metabolic read-outs of hormesis (Calabrese and Blain, 2005), the shape and magnitude of responses, the phenotypes triggered, and the pre-conditioning nature of both processes (Calabrese, 2016), it is therefore acceptable to categorize POS as an example of physiological conditioning hormesis (Calabrese et al., 2007). Mechanistically, we reasoned that mild oxidant production during hypoxia would acts as signaling chemicals that “prepare” animals to stronger redox challenges during re-oxygenation.

## Final statements

It has been previously proposed that the up-regulation of endogenous antioxidants in response to low oxygen exposure in hypoxic-tolerant species could be considered as cases of hormetic responses (Costantini, 2014). This was proposed in a time of uncertainty of (i) how widespread the POS-response in the animal kingdom was, and (ii) on the POS molecular mechanism. We recently demonstrated that 60–70% of all animal species analyzed for antioxidant responsiveness during low oxygen stress or estivation turned to be POS-positive cases (Moreira et al., 2016, 2017). Costantini (2014) proposed that “*reactive species might work as molecular mediators of such hormetic effects*,” referring to the induction of antioxidant enzymes under hypoxia or related situations. Relatively recent works showing activation of redox-sensitive transcription factors in animals under low oxygen stress (Malik and Storey, 2009, 2011; Krivoruchko and Storey, 2010, 2013; Hermes-Lima et al., 2015) are the best indications that RS—including not only oxygen-derived RS, but also aldehyde products of lipid peroxidation (Hermes-Lima et al., 2015)—are the primary mediators of the hormetic effects. Thus, it is reasonable to postulate that conventional mediators participate in the signaling responses triggered by mild stress conditions in the mechanistic framework for POS/hormesis as shown in Figures 1A,**B**. However, a deeper mechanistic understanding of both processes is required to validate POS as a novel example of physiological conditioning hormesis.

Similar to other hormetic phenomena, a threshold of environmental oxygen concentration seems to determine the magnitude of POS response. Such threshold is likely to be species and tissue dependent, given the observed variability of responses. One example is the crab *Neohelice granulata* from Brazilian saltmarshes, which up-regulate glutamate cysteine ligase (GCL), and GPX activities by 53 and 100%, respectively, upon severe hypoxia (Geihs et al., 2013, 2016). However, when the same species was exposed to higher oxygen levels, no alterations in antioxidant enzymes were observed (Leidens, 2017). Conceivably, the mechanisms involved in POS/hormesis are differently expressed or activated by distinct strengths among tissues and species, adding another layer of complexity in the adaptive protective processes to minimize redox damage (Geihs et al., 2014). Finally, assuming that hormetic responses seem more robust when taking place early in life rather than in adult stages (Costantini, 2010; Costantini et al., 2012, 2014), it is conceivable that POS-effects would follow this trend.

Lastly, depending on the magnitude and length of low environmental oxygen levels, the profiles and mechanisms that confer tolerance to hypoxic stress through POS are strikingly similar to those observed in typical hormesis phenomena. For this reason, we postulate herein that POS can be considered an example of physiological conditioned hormesis. In order to take POS research to the next level we should (i) further understand the molecular mechanisms controlling the hormetic POS phenomena, for example providing evidence for the role of kinases/phosphatases in the improvement of the antioxidant response in hypoxic tolerant species, and (ii) keep searching for the POS-response in nature (Moreira et al., 2017) having in mind the heterogeneity/diversity of POS phenotype among distinct tissues and animal species. Importantly, when pursuing for POS in nature, we must be aware of the ecological dimensions of the problem and consider the adaptive value of POS in the field. For example, blue mussels living in upper shores of a rocky intertidal coast, which are more exposed to air-exposure stress, exhibit higher activity of antioxidant enzymes than those from lower shores (Letendre et al., 2009). This example underscores the importance of POS as an adaptive mechanism to cope with environmental challenges, which depends not only on the magnitude of the physiological response, but also on how efficient this response is to deal with stresses generated in a particular environment. Therefore, such “POS eco-research” must consider reproductive stage, feeding conditions, age, and (epi)genetic factors of individuals, as well as abiotic micro-environmental variations in habitats and the potential interactions of all these elements (Vaiserman, 2011; Costantini et al., 2012, 2014).

## Author contributions

MO, DM, and MH-L worked on the concept of hormesis and its application on the POS theory. DM, TF, and MG contributed with key examples of POS-related studies. MO and MH-L drafted the manuscript, which was reviewed and approved by all authors.

### Conflict of interest statement

The authors declare that the research was conducted in the absence of any commercial or financial relationships that could be construed as a potential conflict of interest.
